# Exercise Collapse Associated With Sickle Cell Trait: Diagnosis, Treatment, Prevention, Current Controversies, and Gaps in the Literature

**DOI:** 10.7759/cureus.111737

**Published:** 2026-06-29

**Authors:** Jordan Lyons, Yao-Wen E Hu, Glen A Cook

**Affiliations:** 1 Family Medicine, Uniformed Services University of the Health Sciences, Bethesda, USA; 2 Family Medicine, First Marine Regiment, Camp Pendleton, USA; 3 Family Medicine and Community Health, University of Massachusetts Chan Medical School, Worcester, USA; 4 Neurology, Walter Reed National Military Medical Center, Bethesda, USA; 5 Neurology, Uniformed Services University of the Health Sciences, Bethesda, USA

**Keywords:** exercise collapse associated with sickle trait, exertional sickling, hemoglobin, sickle, sickle cell trait

## Abstract

Sickle cell trait (SCT) is often benign but can be associated with occasional hemoglobin sickling, particularly in the setting of physical exertion. Screening for SCT, while controversial, reduces exertional events and death. Exertional sickling (ES) is the presumed and hypothesized etiology of collapse and death in athletes, where sickling, microvascular occlusion, and muscle necrosis result in ischemia, hyperkalemia, arrhythmia, and fulminant rhabdomyolysis. Due to the lack of clarity surrounding ES, the term exercise collapse associated with SCT (ECAST) was developed to describe individuals with SCT who suffer from an exertional collapse. Certain environmental and physical conditions may increase the likelihood of ECAST, but prompt recognition and treatment may reduce morbidity and mortality. This narrative review identifies current evidence regarding ECAST and discusses treatment, prevention, recent controversies, and gaps in the literature, highlighting that knowledge of SCT among athletes, warfighters, and their supervisors remains inadequate.

## Introduction and background

Sickle cell trait (SCT) is a genetic mutation involving hemoglobin, a protein composed of two alpha (α) and two beta (β) subunits working together to transport oxygen in red blood cells (RBC). Normal hemoglobin with two alpha and two beta subunits allows for proper hemoglobin function, RBC shape (biconcave discoid), and RBC flexibility as it passes through capillary beds [1,2]. A specific position-six point mutation in the hemoglobin beta subunit (HBB), substituting valine for glutamic acid, produces hemoglobin S (HbS), and the inheritance of one normal HBB and one sickle HBB produces SCT [1]. This mutation causes between 30% and 45% of RBCs to contain abnormal hemoglobin but is more stable compared to sickle cell disease (SCD), which results from two mutated HBBs [1,3,4].

SCT affects approximately three million Americans and 300 million individuals globally [5], specifically one in 12 Black Americans and up to three in 10 sub-Saharan Africans, with other ethnic groups affected to a lesser degree [6-8]. In the United States, screening for SCD has been available since the 1970s through newborn screening programs administered by individual states, which were variable until 2006 when national screening was implemented and supported by the US Preventive Services Task Force (USPSTF) [9,10]. Despite guidance, state-dependent screening still resulted in discordant reporting and follow-up of abnormal results, leading to gaps in awareness, diagnosis, intervention, and understanding among patients and communities. Thus, exercise collapse associated with SCT (ECAST) and its suspected pathophysiologic precursor, exertional sickling (ES), are both under-represented in research and clinical practice and misunderstood in the affected communities [11-13]. This narrative review evaluates the current evidence regarding ECAST evaluation, diagnosis, treatment, and prevention, with special focus on current literature gaps, controversies, and future directions.

Review approach and sources

Guidelines from the Preferred Reporting Items for Systematic Reviews and Meta-Analyses extension for searching (PRISMA-S) were used to conduct a comprehensive search of the literature, but as a narrative review, the effort was not exhaustive in the manner of a systematic review. Rather, the intent of the manuscript was to summarize key themes and foundational concepts while identifying recent controversies and gaps in the literature. Sources were identified through focused searches in PubMed, Google Scholar, Medline, and Cochrane databases, using the search terms "exercise collapse associated with sickle cell trait", "exertional sickling", "exertional collapse", "sickle trait", and "sickle exercise." Cited references of sentinel papers in the field, along with published guidelines from the Uniformed Services University (USU) Consortium for Health and Military Performance (CHAMP), the Warrior Heat and Exertion-Related Events Collaborative (WHEC), and related military instructions and protocols were also examined. No other study registries or search methods were used. Searches were limited to the English language, while editorials, letters to the editor, and commentaries were excluded, although there were no restrictions on date of publication due to the perceived gaps in the literature. The initial search yielded 211 manuscripts whose titles and abstracts were reviewed and screened by the authors (JL, YEH, GC) and contributors (JS, JW) for relevance and duplicate records. Full-text reviews of 114 manuscripts were performed by the authors and the contributors, with the final 60 references guided by professional judgment to include literature that shapes the understanding of the topic, focusing on articles published in peer-reviewed journals within the last 50 years.

## Review

Exertional sickling (ES) versus collapse associated with SCT (ECAST)

SCT is typically asymptomatic, but RBC sickling may present under decreased oxygen tension during exercise [14]. ES occurs under repeated bouts of hypoxia during extreme exertion and at altitudes greater than 5,000 feet [8,15]. Additional contributors, such as increased ambient heat index, dehydration, increased blood viscosity from RBC sickling, and inflammation, may contribute to more severe outcomes of sickling, but the significance of these factors is not fully defined [16], though several cases report exercise-related conscious collapse, rhabdomyolysis, tissue hypoxia, and multi-system organ failure in those with SCT [8,17-19]. Harmon et al. showed, in an observational study, that, while the relative risk for exertional death in athletes with SCT versus those without is notable (15 times), the absolute risk remains small (1:4,706), albeit with catastrophic outcomes [20].

ES is the presumed and hypothesized etiology of collapse and death in athletes with SCT, where RBC sickling, microvascular occlusion, and muscle necrosis result in ischemia, hyperkalemia, arrhythmia, and fulminant rhabdomyolysis [20,21]. While ES presents a reasonable pathophysiology to explain increased exertional death rates or the mechanism of collapse in those with SCT, it has yet to be proven, nor is it fully understood [21-23], as there are only rare case reports of myonecrosis during presumed vaso-occlusive episodes in those with SCD [24]. Accordingly, a summit consisting of international experts from various relevant and interested specialties in 2011 adopted the term ECAST in the absence of a completely understood mechanism for collapse, to validate the epidemiology of collapse [16]. However, this summit and others critically noted that the exact pathophysiology of sickling and the subsequent collapse observed in ECAST events are still active areas of controversy and need to be recognized by members of the medical community [16,21-23,25].

Clinical evaluation

ECAST commonly presents in the midst of sustained high-intensity drills, typically during pre-season practice, with an associated "heroic effort" to push to supramaximal levels [26,27]. Symptoms include leg, muscle, or abdominal pain with eventual inability to continue exercise [26,27]. Unique features seen in ECAST include "slumping" to the ground, muscle weakness greater than pain, conscious collapse, normal muscle examination, tachypnea, and rectal temperature less than 104 degrees Fahrenheit [28]. These findings help differentiate ECAST from other conditions, such as exercise-associated muscle cramping, exertional heat stroke (EHS), exertional rhabdomyolysis (ER), and cardiac arrhythmias [28-30] (Table 1). Risk factors associated with ECAST include dehydration, deconditioning, high altitude, elevated ambient temperatures, medications (e.g., diuretics and psychiatric medications), drugs (e.g., cocaine and amphetamines), or supplements (e.g., caffeine) [28]. While these factors appear to increase the risk of ECAST, cases may still occur in the absence of risk factors. The intensity of the event remains the most important clinical clue [29], as collapse can occur within minutes of high-intensity exercise [19,29,31]. Unfortunately, inexperienced coaches and observers may attribute symptoms to deconditioning or exercise-associated muscle cramps.

**Table 1:** Differential diagnosis of exertional collapse - signs, symptoms, and initial treatment. CNS: central nervous system; EMS: emergency medical services; CPR: cardiopulmonary resuscitation; AED: automated external defibrillator; IV: intravenous; POC: point-of-care; O2: oxygen; WNL: within normal limits; TTP: tender to palpation; U/A: urinalysis; RBC: red blood cell; TACO: tarp-assisted cooling oscillation; CHAMP: Consortium for Health and Military Performance; WHEC: Warrior Heat and Exertion-Related Events Collaborative Notes: 1. HE is distinguished from EHS by the absence of CNS and end-organ damage. Temperatures >41°C (105.8°F) should initially be considered as EHS given the possibility of a lucid interval, and that proteins denature at temperatures >41°C (105.8°F), thus resulting in end-organ damage. 2. Simple cooling: ice packs, ice towels/sheets, fan, forearm immersion, etc. 3. Active cooling: ice water immersion, Quantico method, TACO, ice sheets with ice packs. 4. Exercise-associated collapse is also identified as exercise-associated postural hypotension (EAPH). Source: Ref [29]. Permission for use and reprint obtained from the CHAMP/WHEC Medical Director.

Condition	Core Temp	CNS Dysfunction	Timing	Muscles	Urine	Shortness of Breath	Initial Treatment
Sudden Cardiac Arrest (SCA)	Variable	Abrupt loss of consciousness	Variable	Flaccid or seizing	N/A	Apneic or agonal breathing	Activate EMS, CPR, early AED
Exertional Heat Exhaustion (HE)	≥38°C (100.5°F), ≤41°C (105.8°F). See note 1	No	Late	May have exercise-associated muscle cramps (EAMC)	Often dehydrated	Possible	Remove excess clothing, oral fluids (+/– IV fluids), simple cooling, rest. See note 2
Exertional Heat Stroke (EHS)	≥40°C (104.0°F)	Yes (may be intermittent or subtle)	Late	May have EAMC	Possibly dehydrated	Possible	COOL FIRST, COOL FAST, COOL on site +/– IV fluids. See note 3
Exercise-Associated Hyponatremia (EAH)	Usually <39°C (102°F)	Yes, if severe. Often headache, confusion, and/or repeated emesis	Usually late	N/A	Urination may be increased or decreased	No	Oral sodium solution, POC testing, and administer 3% saline if CNS dysfunction or cannot swallow
Exertional Collapse Associated with Sickle Cell Trait (ECAST)	Usually <39°C (102°F). If >40°C (104°F), must treat as EHS	Typically, intact CNS function, “conscious collapse”	Often early	Typically, flaccid lower extremity muscle groups, global pain	May be dark/bloody	Possible, may be severe	O2, AED, IV fluids, activate EMS
Exertional Rhabdomyolysis (ER)	WNL	No	Late	May be stiff and swollen; TTP; pain with active motion	May be dark, “cola-colored” U/A + blood, but no RBCs	No	Rest, oral vs IV fluids
Exercise-Induced Hypoglycemia	WNL	Yes	Late	WNL	Variable	No	Glucose
Exercise-Associated Collapse (EAC). See note 4	WNL	+/– CNS dysfunction that recovers quickly (<15 min)	Late, typically end of event	WNL	Variable	No	• Rule out other causes • Place patient supine with legs elevated 12–24” above the heart • If not rapidly better, re-check for other causes

Initial evaluation of the collapsed athlete includes assessment for responsiveness, airway, breathing, and circulation. Event description should be obtained through direct observation, either via the medical team or bystanders. Physical examination includes vital signs with core temperature, cardiopulmonary evaluation, and focused evaluation of affected areas for affected limb range of motion, strength, neurovascular status, swelling, tenderness, compartment rigidity, and muscle fasciculations to distinguish ECAST from other confounding conditions [32]. Serial assessment for resolution or progression of symptoms is recommended, but intravenous fluids, supplemental oxygen, and transfer to the emergency department should not be delayed, particularly if symptoms progress or if inadequate pulses or respirations are noted [28]. Differential diagnosis for collapsed athletes is broad and may present challenges for first responders [29]. Individuals also may not be aware of their own sickle cell status, understand its implications, or associate SCT with their current symptoms since universal newborn screening for sickle cell status was not instituted until 2006 [10].

Diagnosis

Diagnosis of ECAST is difficult as its findings may mimic a spectrum of conditions ranging from exercise-associated muscle cramps to sudden cardiac death [29,30]. Key differences between ECAST and other confounding conditions, such as EHS and ER, are its early onset, lucid mental status, normal core temperature, progressive weakness more than pain, and normal muscle examination [28,32]. Awareness of SCT status may improve first responder responsiveness to ECAST, but high clinical suspicion, clinical history, and physical examination remain pivotal for timely diagnosis. Laboratory testing may demonstrate multi-organ dysfunction, such as abnormal creatinine, elevated transaminases, increased blood glucose, electrolyte imbalances, and decreased urine output with otherwise noncontributory diagnostic imaging [17,32]. Although ECAST may be associated with ER and elevated creatine kinase (CK) levels, it may also present with physiologic levels of CK after exertion [17,29,32]. Screening for SCT remains controversial yet is currently mandated by all branches of the United States military, including the Space Force, and the National Collegiate Athletics Association (NCAA) [33]. Despite its catastrophic consequences, ECAST is still rare among those with SCT, and the vast majority may still exercise at high intensity [34].

ECAST is commonly attributed as the primary cause of death only after autopsy. However, sickling observed at autopsy may be due to postmortem hypoxia from other causes, further complicating the true connection between SCT and death [35]. Recent controversy around ECAST diagnosis and pathophysiology raised questions about the legitimacy of acute vaso-occlusive sickle cell crisis as a primary cause of death in SCT, especially without ER [21-23]. However, the risk of exertion-related death in SCT is not minimal, and ER rarely results in death when SCT is not present [36]. Lack of awareness for ECAST may contribute to misdiagnosis and delays in treatment, critical missteps since clinical decline may be rapid [17,27]. Cause of death is often attributed to complications from other conditions and not from ECAST itself, as RBC sickling may be unrecognized or underappreciated as an inciting event [23,25,28]. Furthermore, the literature on sudden death during exertion details an expansive list of potential confounding diagnoses, including exertional heat illness, ER, and others. Accurate diagnosis relies on thorough assessment of the athlete while retaining awareness of and suspicion for ECAST in the appropriate context [26].

Treatment

**Figure 1:**
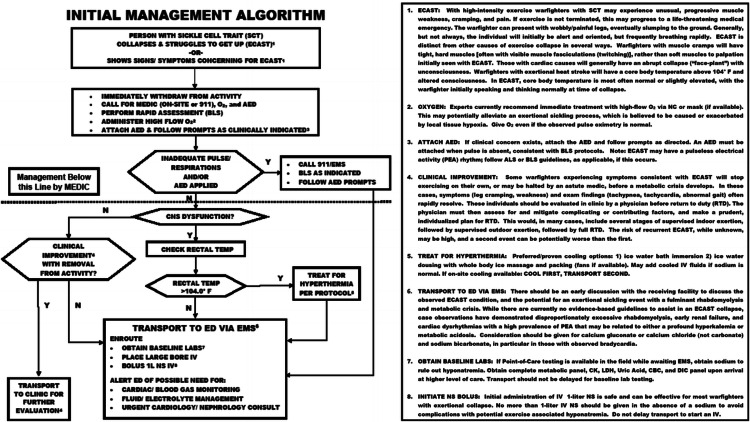
Treatment management algorithm for ECAST or collapsed warrior athlete. O2: oxygen; AED: automated external defibrillator; BLS: basic life support; EMS: emergency medical services; CNS: central nervous system; ED: emergency department; IV: intravenous; NS: normal saline; NC: nasal cannula; ALS: advanced life support; CK: creatine kinase; LDH: lactate dehydrogenase; CBC: complete blood count; DIC: disseminated intravascular coagulation Source: Ref [28]. Permission for use and license for reprint obtained from Wolters Kluwer Health, Inc. (CC-BY License 6270271075173).

ECAST treatment guidelines are primarily based on expert opinion, observational, or retrospective studies. A summit of international experts and interested parties in 2019 proposed clinical practice guidelines and algorithms to care for those with ECAST [28] (Figure 1). Immediate actions to consider in the event of collapse include withdrawing from the inciting activity, assembling trained healthcare providers, administering high-flow supplemental oxygen, and performing basic life support (BLS) with automated external defibrillator (AED) application. Appropriate emergent treatment for other concomitant conditions, such as hyperthermia, should also be considered prior to transport to the emergency department, especially for those who are not improving clinically, hyperthermic, and/or unresponsive [28]. However, in resource-poor settings, access to emergency services, equipment availability, or provider training may not be feasible.

Specifically, timely administration of supplemental oxygen and intravenous fluids may prevent exacerbation and/or reverse the sickling process while treating metabolic acidosis and improving blood flow [28,37]. Oral fructose-containing fluids may also be considered an early alternative where intravenous access is not immediately obtainable, as studies have shown in vitro reduction of hypoxia and restoration of near normal blood flow despite low oxygen levels [38]. Exertional collapse, ECAST, and any other concurrent conditions resulting in medical intervention should serve as a warning for future events, raising clinical suspicion for subsequent recurrences.

Prevention

NCAA Division I football players with SCT demonstrate a 37-fold increase in exercise-related death (ERD) compared to athletes without SCT in an observational study [20]. Risk reduction focuses on primary, secondary, and tertiary prevention via screening, education, and return-to-play guidelines, respectively.

Screening

Screening for hemoglobinopathies has been recommended by the National Institutes of Health Consensus Development Conference on Newborn Screening for Sickle Cell Disease and Other Hemoglobinopathies since 1987, with all states and the District of Columbia adopting screening in 2006 [6,9]. In 2008, the U.S. Preventive Services Task Force (USPSTF), with high certainty, endorsed substantial net benefit for newborn sickle cell screening [10], while in 2010, the NCAA implemented a policy to determine sickle cell status through testing or historical review in Division I athletes [33] and extending it to Division II and III athletes in 2012 and 2014, respectively [39]. However, athletes may still opt out of testing until 2022, when determination became mandatory for all collegiate athletes [33]. Historically, individual branches of the United States military separately decided whether to perform SCT screening; screening may occur at different points of military service and was only required for high-risk service members based on training or deployment risk [34,40]. However, as of 2020, all branches of the military, with the Space Force falling under the Air Force umbrella, perform universal SCT screening at service entry [34]. Despite its demonstrated benefit, the ethics of mandatory SCT screening are still debated [8]. Institutions need to balance population-level safety objectives with individual autonomy and choice. Forced testing and screening may potentially violate personal freedoms while introducing potential stigma, especially in an environment built on the appearance of strength. Regardless of the screening results, the perception of medical testing may project weakness and inability to support the mission and goals of the collective team, leading to delays in medical treatment or declining psychological health.

Education

Knowledge and awareness of SCT amongst athletes, coaches, trainers, and medical staff remain inconsistent even though screening is often recommended. Only 16% of individuals and 37% of their parents are aware of their SCT diagnosis [41,42]. These gaps hinder prevention, diagnosis, and treatment, which in turn increases the risk of catastrophe. Athletes and family members should be informed of the diagnosis and potential complications related to SCT. While exercise is not typically restricted, discussion of warning signs and red flags may encourage withdrawal from exercise prior to collapse. Factors such as prior collapse, level of conditioning, hydration, asthma, recent illness, comorbid cardiac conditions, and certain medications all potentially increase the risk of ECAST [28]. Furthermore, environmental factors, including ambient temperature, elevation above sea level, and lack of acclimatization, may elevate risk for ECAST [16,28]. Athletes should be mindful of these particular risk factors and advocate for their own health and safety.

Physicians and healthcare professionals should be keenly aware of the possible complications of extreme exertion in those with SCT. Medical providers should educate patients, parents, coaches, trainers, and other appropriate personnel about common signs and symptoms of ECAST, along with triggers for medical care. Those with SCT are also at increased risk for splenic infarction, renal necrosis, and pulmonary embolism, all of which can present with exertional collapse [37]. Following appropriate treatment algorithms, such as those proposed from the second ECAST summit in 2019 (Figure 1), contacting the receiving medical facility, communicating concerns with the receiving medical treatment team, and keeping ECAST high on the list of differential diagnoses, lead to decreased morbidity and mortality [28], as decompensation, profound rhabdomyolysis, and death may occur rapidly. Adherence to return-to-play guidance, along with continued close clinical follow-up, is important since minor incidents may present as sentinel events for future recurrence and catastrophe [28].

The coaching staff, strength and conditioning team, and athletic trainers should also be familiar with concerns related to extreme exertion, risks of environmental factors, individual conditioning statuses, and the presentation of ECAST. Many athletic events, practices, drills, and scrimmages are conducted without qualified medical personnel present. Consideration for environmental factors, including ambient temperature, elevation, hydration, and rest-work cycles during practice, conditioning, and competition, is pivotal [28]. Knowledge of SCT status, ECAST, and its related complications facilitates advocating for safety instead of powering through weakness and fatigue, common precursors for impending collapse [32]. Understanding and support of the struggling athlete, rather than negative reinforcement, prevents catastrophe.

Return to Play

**Figure 2:**
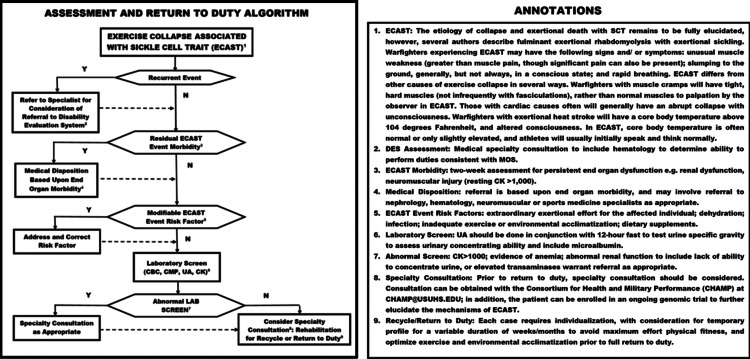
Assessment and return-to-duty algorithm in warrior athletes with ECAST. CBC: complete blood count; CMP: comprehensive metabolic panel; UA: urinalysis; CK: creatine kinase; SCT: sickle cell trait; DES: disability evaluation system; MOS: military occupational specialty Source: Ref [28]. Permission for use and license for reprint obtained from Wolters Kluwer Health, Inc. (CC-BY License 6270271075173).

Currently, no evidence-based guidance exists for return to activity after ECAST [32]. However, proposed return-to-play algorithms (Figure 2) for ECAST may serve as initial guidance for clinical providers [28]. As these algorithms and recommendations are largely consensus-based, clinical judgement and individual assessment remain essential until prospective validation studies are available. SCT correlates with renal necrosis, splenic infarction, pulmonary thrombosis, and explosive rhabdomyolysis [37]. Although they may present concurrently with ECAST, each condition deserves thorough investigation, resulting in individualized and holistic approaches for treatment and return to activity [28]. Discussion of symptoms and medical care with involved individuals provides education for potential future events. In the absence of recurrence, residual ECAST morbidity, and modifiable risk factors, proposed return-to-play guidelines call for laboratory testing, including complete blood count, comprehensive metabolic panel, urinalysis, and CK to screen for possible underlying and concomitant medical conditions [28]. Appropriate treatment for other medical conditions adds to the highly individualized protocols for return-to-play. Specialty consultation is highly recommended in the face of recurrent ECAST and positive laboratory testing to assist with not only treatment but also return-to-play guidelines [27,28]. Nonetheless, graded return to activity with appropriate supervision limits the risk of recurrence and catastrophe [32].

Gaps in the literature

Scientific understanding of SCT, ES, and ECAST has advanced significantly, but critical gaps in the literature continue to hinder effective prevention, diagnosis, and treatment. These gaps span multiple domains, including pathophysiology, screening, treatment, forensic evaluations, and public health initiatives.

Pathophysiology Research Gaps

Although the role of blood flow dynamics and oxidative stress in SCT is well-documented, the molecular mechanisms driving these changes remain poorly understood. While upregulation of intercellular adhesion molecule-1 (ICAM-1) and vascular cell adhesion molecule-1 (VCAM-1) for increased RBC adhesion to endothelium is observed, specific triggers during exercise remain unknown [43,44]. Similarly, the impact of oxidative stress and NO depletion in SCT carriers, particularly during post-exercise recovery, requires further research [45]. Genetic modifiers, such as α-thalassemia and specific single-nucleotide polymorphisms (SNP), influence the severity of SCT-related complications, but their exact mechanisms are uncertain [46,47]. Future large-scale genomic studies clarifying the role of these modifiers and identifying potential genetic risk factors are needed. However, emerging therapeutic agents, such as GBT440, have demonstrated prevention of RBC sickling under strenuous exercise in vitro via increased oxygen affinity and inhibition of hemoglobin polymerization [48]. Further research is needed to validate in vivo findings and to establish safety and efficacy.

Screening and Diagnostic Challenges

SCT screening varies widely across institutions with debatable effectiveness. Both ECAST summits in 2011 and 2019 highlighted the drawbacks of mandatory screening, noting potential for stigma and limited predictive value of isolated testing [16,28]. Although the NCAA's mandatory screening reduced ECAST fatalities, questions persist regarding cost-effectiveness and ethical implications [32,49-51]. Reliance on clinical judgment also creates variability for diagnosing ECAST, as symptoms often overlap with those of EHS, ER, and cardiac conditions [11,29,52]. Identifying reliable biomarkers for early detection of ECAST, especially for oxidative stress and vascular adhesion, could revolutionize diagnosis and enable targeted interventions for at-risk individuals, but further research is needed [43,52].

Treatment and Prevention Research Gaps

Despite growing awareness of ECAST, evidence-based treatment protocols remain scarce. Current management strategies, such as hydration and oxygen supplementation, are based on theory rather than empirical data [52,53]. Both ECAST summits in 2011 and 2019 emphasized the need for validated clinical guidelines to guide acute and long-term management of ECAST and its complications [16,28]. Further research is needed to evaluate the efficacy of interventions and to develop validated, comprehensive guidelines for both acute management and long-term prevention.

With respect to prevention, light activity and active post-exercise recovery have been proposed as methods to mitigate post-exercise complications, but further research is needed to validate these approaches [19,52]. Challenges for prevention arise from a lack of predictability and a poor understanding of temporal and acute risk in the specific moment. Many athletes with SCT train and compete at high intensities without incident, but others experience ECAST episodes further into their careers [22]. Prevention in the United States military is also particularly critical, as ECAST in this population often occurs under extreme exertion and austere environments. Current data suggest that sustained all-out exertion is the primary trigger for ECAST, with heat and dehydration acting as additional variables [17,31,54]. However, standardization and strategies for tailoring preventive measures are difficult due to the unique demands and requirements of individual military services [37,55].

Forensic Research Gaps

Whereas international organizations, such as the Royal College of Pathologists, established guidelines for autopsies, the lack of standardized autopsy protocols within the United States hinders forensic evaluations for ECAST-related fatalities [56]. Current practices vary widely, leading to inconsistent reporting and potential underestimation of ECAST incidence and prevalence [44,57,58]. Uniform guidelines for the examination and documentation of ECAST-related deaths improve diagnostic accuracy, consistency, and generalizability of epidemiologic data to the population as a whole [16,28,57]. Integrating genetic analyses into routine autopsy protocols to identify relevant mutations, such as LAMA2, in ECAST fatalities may uncover otherwise overlooked factors, providing insights into individual susceptibility [16,47,59].

Public Health and Education Gaps

Public health initiatives addressing SCT highlight significant gaps in education and outreach. Studies indicate that knowledge and awareness among athletes is alarmingly low, with only 16% of athletes aware of their own SCT status [41]. Much of the literature focuses on high-performance athletes (i.e., NCAA, military), leading to successful screening and improved outcomes. However, it is important to promote consistency and generalizability across all individuals engaging in exercise. Education for recreational athletes, the general population, and healthcare providers expands understanding and awareness of SCT and ECAST, particularly regarding their risks during extreme exertion [16,28]. Additionally, most research focuses on acute complications and leaves long-term health implications and chronic effects of SCT and ECAST unaddressed, further limiting the ability for comprehensive counseling and management strategies [5,37,60]. Addressing these literature gaps requires collaborative research across clinical, forensic, and public health domains. Prioritization of research in pathophysiology, screening, and prevention may lead to targeted strategies, improved outcomes, and reduction of complications.

Current controversies

Recent literature renewed controversy in SCT risk, screening, and ECAST identification. Carney et al. concluded that SCT screening may result in inequitable and possibly harmful treatment in collegiate athletes and the military, proposing that SCT is not associated with a higher risk of death but rather a higher risk of ER [25]. Weeks et al. suggested that there are no data to support acute vaso-occlusive sickle cell crisis as a cause of death in SCT, nor does evidence support SCT as a cause for exertion-related death without rhabdomyolysis [23]. These publications oppose current expert opinion and data that not only does SCT increase the risk of exertion-related death [20,57], but there is also increased relative risk for death even without ER [35]. However, it is important to note that much of the currently available evidence is observational. Nonetheless, there is additional evidence that screening, education, and prevention are not harmful and are potentially life-saving [51].

## Conclusions

ECAST is a complex condition that poses a significant risk under physiological and environmental duress. Acknowledgement of SCT diagnosis, prompt on-site recognition of ECAST symptoms, and targeted, timely interventions are essential to increasing awareness and risk mitigation. Addressing inequity and controversy directly with integration of research, adherence to clinical practice, and development of public health initiatives potentially enhances prevention, diagnosis, and treatment, with goals of decreasing morbidity and mortality.
